# Research to Optimize the Action and Impact of Local Age-Friendly Community Efforts

**DOI:** 10.1093/geroni/igaf122.1691

**Published:** 2025-12-31

**Authors:** Mildred Warner, Natalie Pope, Bill Armbruster

## Abstract

This symposium features four empirical studies that explore how local-level age-friendly community (AFC) action and impact are created using a range of methodological approaches to understand the experiences and perspectives of AFC leaders across diverse settings. The first two papers use survey methods, with one focused on examining the role of mobility managers for advancing transportation alternatives as part of AFC progress in Ohio. Results highlight the importance of training and education, regional planning, and inter-organizational collaboration to amplify impact. The second paper presents findings from a survey of municipal leaders across the country regarding planning, the built environment, and services. Findings indicate age-friendly action is stalled at the municipal level, suggesting that more attention to challenges faced in suburban and rural environments is needed. The third paper presents a study based on qualitative interviews with older adults across four states to identify the developmental strengths they leverage to advance local age-friendly implementation. Findings highlight assets such as their long-standing community relationships, personal experiences of aging, and relevant professional skills. The fourth paper showcases a project that engaged AFC initiative leaders across multiple countries to create a novel assessment tool to measure the impact of AFC initiatives in terms of “spillover” or unexpected positive outcomes from local AFC efforts. Discussant remarks will expand on the importance of promoting age-friendly praxis wherein knowledge (production) and practice dynamically advance insights into the contexts, facilitators, processes, and outcomes of local-level age-friendly work to strengthen the movement in the U.S. and worldwide.

